# Molecular profiling of long‐term responders to immune checkpoint inhibitors in advanced non‐small cell lung cancer

**DOI:** 10.1002/1878-0261.12891

**Published:** 2021-01-06

**Authors:** Joan Frigola, Alejandro Navarro, Caterina Carbonell, Ana Callejo, Patricia Iranzo, Susana Cedrés, Alex Martinez‐Marti, Nuria Pardo, Nadia Saoudi‐Gonzalez, Debora Martinez, Jose Jimenez, Irene Sansano, Francesco M. Mancuso, Paolo Nuciforo, Luis M. Montuenga, Montse Sánchez‐Cespedes, Aleix Prat, Ana Vivancos, Enriqueta Felip, Ramon Amat

**Affiliations:** ^1^ Thoracic Cancers Translational Genomics Unit Hebron Institute of Oncology (VHIO) Vall d Barcelona Spain; ^2^ Oncology Department Vall d’Hebron University Hospital & Vall d’Hebron Institute of Oncology (VHIO) Barcelona Spain; ^3^ Department of Medical Oncology Hospital Clinic Barcelona Spain; ^4^ Translational Genomics and Targeted Therapies in Solid Tumors IDIBAPS Barcelona Spain; ^5^ Molecular Oncology Group Vall d'Hebron Institute of Oncology (VHIO) Barcelona Spain; ^6^ Pathology Unit Vall d’Hebron University Hospital Barcelona Spain; ^7^ Cancer Genomics Laboratory Vall d’Hebron Institute of Oncology (VHIO) Barcelona Spain; ^8^ Program in Solid Tumors Center for Applied Medical Research (CIMA) Pamplona Spain; ^9^ Department of Pathology, Anatomy and Physiology School of Medicine University of Navarra Pamplona Spain; ^10^ Centro de Investigación Biomédica en Red de Cáncer (CIBERONC) Madrid Spain; ^11^ Navarra Health Research Institute (IDISNA) Pamplona Spain; ^12^ Cancer Genetics Group Josep Carreras Leukaemia Research Institute (IJC) Campus ICO‐Germans Trias i Pujol Badalona, Barcelona Spain

**Keywords:** chromosomal alterations burden, copy number alterations, immune checkpoint inhibitors, long‐term benefit, NSCLC, PD‐L1, tumor mutational burden

## Abstract

Immunotherapy has transformed advanced non‐small cell lung cancer (NSCLC) treatment strategies and has led to unprecedented long‐lasting responses in some patients. However, the molecular determinants driving these long‐term responses remain elusive. To address this issue, we performed an integrative analysis of genomic and transcriptomic features of long‐term immune checkpoint inhibitors (ICIs)‐associated responders. We assembled a cohort of 47 patients with NSCLC receiving ICIs that was enriched in long‐term responders [>18 months of progression‐free survival (PFS)]. We performed whole‐exome sequencing from tumor samples, estimated the tumor mutational burden (TMB), and inferred the somatic copy number alterations (SCNAs). We also obtained gene transcription data for a subset of patients using Nanostring, which we used to assess the tumor immune infiltration status and PD‐L1 expression. Our results indicate that there is an association between TMB and benefit to ICIs, which is driven by those patients with long‐term response. Additionally, high SCNAs burden is associated with poor response and negatively correlates with the presence of several immune cell types (B cells, natural killers, regulatory T cells or effector CD8 T cells). Also, *CD274* (PD‐L1) expression is increased in patients with benefit, mainly in those with long‐term response. In our cohort, combined assessment of TMB and SCNAs burden enabled identification of long‐term responders (considering PFS and overall survival). Notably, the association between TMB, SCNAs burden, and PD‐L1 expression with the outcomes of ICIs treatment was validated in two public datasets of ICI‐treated patients with NSCLC. Thus, our data indicate that TMB is associated with long‐term benefit following ICIs treatment in NSCLC and that TMB, SCNAs burden, and PD‐L1 are complementary determinants of response to ICIs.

## Graphical abstract

We molecularly characterized tumors from non‐small cell lung cancer patients, focusing on long‐term responders to immune checkpoint inhibitors and showed that these patients present high tumor mutation burden and low somatic copy number alteration burden. PD‐L1 expression was also enriched in these patients. Finally, we validated our findings by reanalyzing two public datasets.

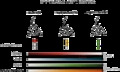

**Abbreviations**
FFPE: formalin‐fixed paraffin‐embedded
FGA: fraction genome altered
HLA: human leukocyte antigen
ICIs: immune checkpoint inhibitors
NSCLC: non‐small cell lung cancer
OS: overall survival
PD1: programmed death 1
PD‐L1: programmed death‐ligand 1
PFS: progression‐free survival
SCNAs: somatic copy number alterations
TMB: tumor mutational burden
WES: whole‐exome sequencing

## 1 Introduction

Lung cancer is the world’s leading cause of cancer‐related deaths due to its high incidence and low survival. Fortunately, immune checkpoint inhibitors (ICIs), antibodies that block programmed death 1 (PD1) receptor or its ligand programmed death‐ligand 1 (PD‐L1), have shown great efficacy leading to responses of unprecedented duration in some patients [1, 2, 3, 4]. Nevertheless, the majority of patients fail to respond to this type of immunotherapy, and many that do eventually develop resistance [5]. Therefore, the identification and validation of biomarkers of ICIs response, and specifically of sustained benefit, are highly relevant to the management of NSCLC patients.

Assessment of PD‐L1 expression by immunohistochemistry is the only validated test for ICIs first‐line treatment decisions in NSCLC [6, 7]. Additionally, PD‐L1 expression has been recently associated with long‐term response to pembrolizumab, an anti‐PD‐1 drug [8]. However, PD‐L1 determination presents limitations such as analysis variability or intra‐tumor heterogeneity. Moreover, favorable responses to ICIs in patients with no apparent expression of PD‐L1 have also been observed. Hence, there is a need to find additional biomarkers or combinations of them to better predict response. In this direction, the quantitative analysis of the presence of different immune cell types infiltrated within the tumor, which can be inferred from gene transcriptional data, has been shown to be indicative of benefit [9, 10, 11, 12, 13].

Tumor mutational burden (TMB) has also been associated with ICIs response in several tumor types such as melanoma and lung cancer [10, 14, 15]. Conceptually, a higher number of somatic mutations increase the amount of potentially immunogenic neoantigens that could be recognized by the adaptive immune system. Nevertheless, the association between TMB and ICIs response has not been observed in other tumor types such as renal cell carcinoma [16]. Moreover, the suitability of TMB as biomarker of ICIs response, especially in melanoma, has been questioned [17, 18]. A less explored feature in relation to ICIs is the tumor’s somatic copy number alterations (SCNAs) burden. In melanoma, high levels of aneuploidy (SCNAs encompassing whole chromosome arms or entire chromosomes) or an overall increase of SCNAs burden were associated with poorer response to ICIs [17, 19], which could be explained by the fact that highly aneuploid tumors exhibit lower levels of immune‐related transcriptional signatures [19]. Conversely, TMB does not seem to correlate with tumor immune infiltration, nor with SCNAs burden or PD‐L1 [8, 11, 20]. Therefore, models combining TMB and PD‐L1 expression or immune‐related signatures have been shown to better predict response to ICIs [11, 20].

Nevertheless, most efforts have been directed toward identifying biomarkers of ICIs response, that is durable clinical benefit (PFS > 6 months), while studies assessing their value in predicting long‐term benefit are scarce, owing to the lack of long‐lasting clinical follow‐up and the low representation of long‐term responders in unselected cohorts. Moreover, studies often focus on just one or two biomarkers. Thus, there is a need for integrative studies analyzing multiple molecular biomarkers in the same set of individuals presenting prolonged response to understand ICI‐associated long‐term benefit. Here, we gathered a cohort of ICI‐treated patients with advanced NSCLC, some of whom exhibited outstanding long‐term responses to these therapies, and assessed tumor‐intrinsic genomic biomarkers and extrinsic biomarkers (immune infiltration) to study their interplay and utility to discern ICIs response and long‐term benefit. Finally, we validated our findings in two publicly available independent cohorts [14, 20].

## 2 Materials and methods

### 2.1 Patients data

All patients included in our cohort were diagnosed with advanced NSCLC and treated at the Vall d’Hebron Hospital. Written informed consent was obtained from all patients before enrollment and the Hospital Institutional Review Board approved this study (PR(AG)139/2014). The study methodologies conformed to the standards set by the Declaration of Helsinki. Detailed clinical information regarding the cohort and each patient can be found in Tables S1 and S2. Archive tumor samples were obtained prior to ICIs treatment. Patients were retrospectively collected.

### 2.2 Whole‐exome sequencing

Whole‐exome sequencing was performed on DNA extracted from formalin‐fixed paraffin‐embedded (FFPE) tumor samples (Maxwell^®^ 16 FFPEPlus LEV DNA Purification Kit, Promega, Madison, WI, USA) in addition to freshly obtained peripheral blood or normal tissue. WES Libraries were prepared according to manufacturer’s protocol (SureSelect XT Human All Exon v5, Agilent, Santa Clara, CA, USA). Finally, libraries were sequenced in a HiSeq2500 (Illumina, San Diego, CA, USA), 2X100 Paired‐end. Reads were aligned against the hg19 reference genome.

### 2.3 Mutation calling

Mutation calling and subsequent filtering were performed using the Mutect2‐GATK pipeline [21]. During the filtering process, cross‐sample contamination was assessed. Those samples with a percentage of cross‐sample contamination greater than 1% were discarded. Resulting mutations were annotated using ANNOVAR [22].

### 2.4 SCNAs calling

Given the difficulties of estimating SCNAs from WES data, SCNAs assessment was performed using two independent methods: CNVkit [23] and Sequenza [24]. CNVkit was run providing the tumor purity estimated by the pathologist as input. In the case of Sequenza, sample purity was estimated by the tool itself. Only regions found to bear SCNAs alterations by both methods were considered for further analysis. Additionally, due to the limitations of assessing SCNAs in low purity samples, those with an estimated tumor purity by Sequenza of < 20 and a tumor purity assessed by the pathologist of < 40 were discarded.

### 2.5 TMB and SCNAs burden

Tumor mutational burden was computed as the sum of all exonic nonsynonymous mutations, insertions, and deletions per sample.

SCNAs burden per patient was computed as the sum of the sizes of all genomic regions affected by SCNAs.

### 2.6 Gene expression assessment

Gene expression by Nanostring was carried out as previously described in Prat et al [13, 25]. In fact, 8 out of 22 samples were previously published in Prat [13]. Nanostring results were then normalized following the Nanostring Gene Expression Data Analysis Guidelines.

General transcriptional signatures associated with T‐cell activity in the tumor were obtained from different publications [9, 12, 19, 26]. Gene expression signatures characteristic of different immune cell populations were obtained from Davoli [19]. Those signatures with less than 50% of the genes represented in the Nanostring PanCancer Immune Profiling Panel were discarded. A list of genes used in each signature is shown in Table S3.

The value of each of these signatures per sample was computed as the geometric mean of the expression values of all genes included in the signature. Next, the values of all samples for each signature were standardized by subtracting the mean value of the signature in the cohort and dividing by the standard deviation.

### 2.7 Statistical analysis

Mann–Whitney Wilcoxon (MWW) tests were performed using the Statannot python library. MWW tests comparing TMB and immune infiltration scores across ICIs benefit groups were left‐sided. MWW tests comparing SCNAs burden across ICIs benefit group were right‐sided. Finally, MWW tests regarding TMB or SCNAs burden differences across clinical features were two‐sided.

Survival analyses were performed using the lifelines python package. Univariate Cox proportional hazards models were built for each feature separately. Also, multivariate models were built combining features of interest. Additionally, multivariate models were stratified by histology and smoking history of the patient.

Variables correlation. The relationship between analyzed features was assessed using both Spearman's rank and Pearson correlations.

### 2.8 Validation cohorts

The Non‐Small Cell Lung Cancer MSKCC cohort (NSCLC‐MSK) [20] was downloaded from cBioPortal [27] (https://www.cbioportal.org/study/summary?id=nsclc_pd1_msk_2018).

Patients with available TMB, FGA (estimation of SCNAs burden), and PD‐L1 expression data were selected and stratified into three groups based on their PFS following ICIs treatment (<6 months, 6‐18 months, or > 18 months of PFS). Patients with a PFS < 18 months but with no confirmed progression (censored) were discarded. Then, TMB, SCNAs burden, and PD‐L1 were compared between groups.

Additionally, TMB, FGA, and PD‐L1 expression was incorporated into a Cox proportional hazards model together with sex, age, and smoking status. Unlike the previous analysis, progression‐free patients with a PFS under 18 months were included. This model was stratified by tumor histology.

A second cohort was used as further validation. Data were downloaded from the supplementary materials of the publication [14]. Patients with both TMB and PD‐L1 data were selected and stratified into three groups based on their PFS as described above. Next, the distribution of TMB and PD‐L1 expression between these groups was compared.

## 3 Results

### 3.1 Patients and clinical setting

We assembled a cohort of 47 patients diagnosed with advanced NSCLC and treated with ICIs as monotherapy. Detailed clinical description is shown in Table S1 and S2. 27 patients exhibited durable clinical benefit defined as a progression‐free survival (PFS) > 6 months, while 20 progressed within this period. Our cohort included 15 patients who exhibited a PFS > 18 months, exceeding 36 months in 8 cases, and 6 of whom remained without signs of progression at the time of closing this study (reaching 60 months in 3 cases).

### 3.2 Tumor‐intrinsic features and response to ICIs

We performed whole‐exome sequencing (WES) from tumor samples of all 47 patients and their paired‐normal sample. After appropriate quality controls, 44 samples were found to be suitable for further analysis. We determined the TMB for these 44 samples and correlated it with different clinical features including histology, sex, smoking status, and drug’s target (Fig. S1). As expected, patients with smoking history presented significantly higher TMB than never smokers (median 387 and 95, respectively; Mann–Whitney–Wilcoxon test (MWW) *P* = 0.004).

**Fig. 1:**
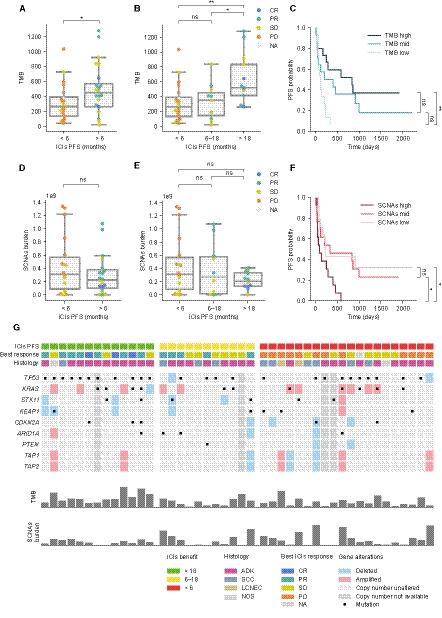
Tumor genomic characterization and response to ICIs. (A, B) Tumor mutational burden (TMB) distribution across groups of ICIs benefit. Color indicates best response to ICIs (CR: complete response, PR: partial response, SD: stable disease, and PD: progressive disease). (C) Kaplan–Meier plot dividing the cohort into TMB tertiles. (D, E) Somatic copy number alterations (SCNAs) burden distribution across groups of ICIs benefit. Color indicates best response. (F) Kaplan–Meier plot survival curves dividing the cohort into SCNAs burden tertiles. (G) Genomic alterations in selected genes. Columns and rows represent patients and genes, respectively. Mann–Whitney–Wilcoxon tests have been used to determine differences between ICIs benefit groups (A, B, D, E). Log‐rank tests have been used to determine differences between TMB groups and between SCNAs groups (C, F). ns: 0.05 < *P* ≤ 1.0; *: 0.01 < *P* <0.05; **: 0.001 < *P* ≤ 0.01.

We next interrogated the association between TMB and response to ICIs. Patients who presented durable clinical benefit (PFS > 6 months) had slightly higher TMB than those with no clinical benefit (MWW, *P* = 0.029) (Fig. 1A). Patients were then stratified into three groups based on their PFS: no benefit (< 6 months), moderate (6‐18 months), and long‐term benefit (> 18 months). When comparing TMB distribution across groups, patients with long‐term benefit had substantially higher TMB than those with moderate or no benefit (MWW, *P* = 0.01 and 0.003, respectively) (Fig. 1B), while no statistically significant differences were observed between these two groups (Fig. 1B). Additionally, Kaplan–Meier analysis of patients stratified into TMB tertiles indicated that patients in the upper tertile had longer PFS than those in the lower one (log‐rank test, *P* = 0.003) (Fig. 1C).

Somatic copy number alterations burden has also been reported to influence ICIs response [17, 19, 28]. To determine its importance in our cohort, we first inferred SCNAs for those samples with enough tumor purity (40 samples, see methodology) and computed the sum of the size of all altered regions of the genome. Then, we related this score to patients’ PFS. No statistically significant differences were observed when stratifying patients into the three categories of benefit defined above, even though we could observe that those with clinical benefit tend to have lower SCNAs burden (Fig. 1D,E). Indeed, Kaplan–Meier analysis dividing patients into tertiles based on their tumor’s SCNAs burden revealed that those in the upper tertile had statistically significant shorter PFS than those in the intermediate and lower tertiles (log‐rank test, *P* = 0.029 and 0.023, respectively) (Fig. 1F). These data suggest that high levels of SCNAs are associated with decreased PFS, while patients with moderate and low SCNAs burden levels present no differences in response to ICIs.

Finally, we assessed whether mutations or copy number alterations in individual genes were associated with response (complete list of somatic alterations Supplementary Tables S4 and S5). After appropriate multiple‐test correction, no alterations were found to be significantly associated with response. We also explored the distribution of somatic alterations in known lung cancer‐related genes and genes associated with ICIs response (Fig. 1G). However, no single gene was found to be significantly enriched in any of the groups (data not shown).

### 3.3 Tumor immune‐related transcriptional signatures and response to ICIs

**Fig. 2:**
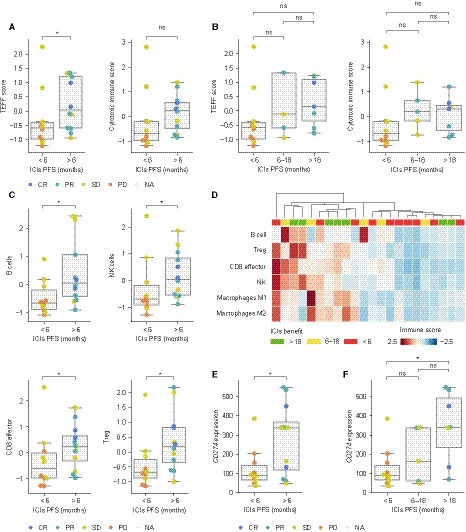
Immune expression profiling of tumor’s microenvironment. (A, B) TEFF and cytotoxic score distribution across groups of ICIs benefit. For each patient, the value of each signature has been computed as the geometric mean of the expression of all genes in the signature. Next, for each of the scores, all values have been standardized. Color indicates best response to ICIs (CR: complete response, PR: partial response, SD: stable disease, and PD: progressive disease). (C) Distribution of expression signatures belonging to four different immune cell populations across groups of benefit. Values computed as in panel A. Color indicates best response to ICIs. (D) Representation of different immune populations across 22 patients. Columns and rows correspond to patients and specific immune cell populations, respectively. Patients have been clustered using a hierarchical clustering algorithm based on their degree of similarity considering amount and type of tumor immune infiltration. (E) *CD274* expression distribution according to indicated group of benefit. Color indicates best response. Mann–Whitney–Wilcoxon tests have been used to determine differences between ICIs benefit groups (A, B, C, E, F); ns: 0.05 < *P* ≤ 1.0; *: 0.01 < *P* <0.05; **: 0.001 < *P* ≤ 0.01.

The amount and type of immune cells infiltrated within the tumor have been suggested to influence response to ICIs [9, 12, 13]. Thus, we obtained RNA expression data from 22 patient’s biopsies by using a Nanostring panel enriched in immune‐related gene transcripts. We evaluated whether transcriptional signatures previously associated with activated T cells were related to ICIs response [9, 12, 19, 26]. Indeed, patients with clinical benefit exhibited higher levels of these signatures compared to those with lack of benefit; some were statistically significant while others were close to significance (TEFF score, MWW, *P* = 0.046) (Fig. 2A, Fig. S2A, S2B). When stratifying patients into the three categories of benefit, no differences were observed between moderate and long‐term benefit (Fig. 2B, Figs S2A, S2B), suggesting that the results in Fig. 2A are not likely driven only by long‐term responders. Of note, this analysis has limited statistical power due to sample size limitations and should be taken cautiously.

Since the Nanostring panel encompasses a broad range of gene transcripts, we analyzed immune cell‐type‐specific signatures [19] and found that B cells, CD8 effective cells, natural killers, and T‐reg were significantly enriched in patients with benefit (MWW, *P* = 0.011, 0.035, 0.035, and 0.014, respectively), whereas M2 macrophages or other scores related to immunosuppressive microenvironments were balanced between both groups or even exhibited a trend toward being higher in nonresponders (Fig. 2C,D, Fig. S2B, S2C).

Finally, we examined *CD274* (PD‐L1) expression from the Nanostring data in these 22 patients and found that high levels of *CD274* expression were associated with clinical benefit (MWW, *P* = 0.040) (Fig. 2E). Notably, stratifying benefit into moderate and long term revealed that high *CD274* expression levels were most evident in long‐term responders compared to patients with no benefit (MWW, *P* = 0.014) (Fig. 2F). It is worth highlighting that for a few patients with Nanostring data, PD‐L1 expression by immunohistochemistry was also available. In those, correlation between *CD274* and PD‐L1 was high (Spearman’s rank correlation, *P* = 0.0002, Fig. S2D).

### 3.4 Interplay between tumor‐intrinsic and tumor‐extrinsic features

**Fig. 3:**
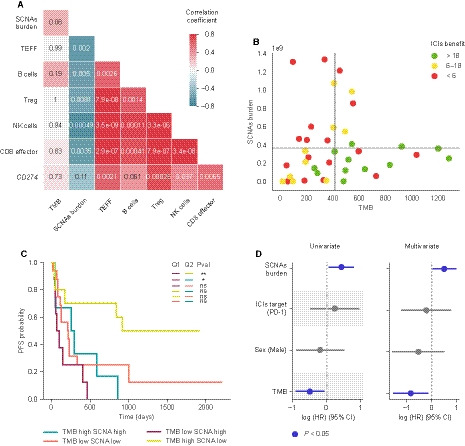
Interplay between biomarkers of response to ICIs. (A) Spearman correlation between indicated features. Color represents the correlation coefficient. The *P* value of each correlation is shown within each cell. (B) Scatter plot representation of TMB and SCNAs burden. Horizontal and vertical gray lines represent the SCNAs burden and TMB mean, respectively. Dot color represents the group of benefit. (C) Kaplan–Meier plot survival curves dividing the cohort into four groups based on the quadrants defined in panel 3B. Long‐rank tests have been used to determine differences between TMB‐SCNAs groups. ns: 0.05 < *P* ≤ 1.0; *: 0.01 < *P* <0.05; **: 0.001 < *P* ≤ 0.01. (D) Univariate and multivariate Cox proportional hazards model for SCNAs burden, ICIs target, sex, and TMB. Multivariate analysis has been stratified by histology and smoking history. Features represented in blue are statistically significant (*P* < 0.05).

To better understand the determinants of response to ICIs, we also studied the interplay between tumor‐intrinsic and tumor microenvironment’s immune features. We found that SCNAs burden negatively correlates with most of the immune‐related transcriptional signatures evaluated but not with *CD274* (PD‐L1) expression, while TMB appeared to be unrelated to all features analyzed (Fig. 3A, Fig. S3A, S3B). As TMB and SCNAs burden are likely independent, we evaluated how their combination related to ICIs benefit. Thereby, we represented patients’ TMB, SCNAs burden, and PFS and then divided the cohort into four groups by using the SCNAs burden and TMB means as thresholds. This analysis revealed that almost all long‐term responders had above‐average TMB and below‐average SCNAs burden, while patients whose tumors present opposite characteristics were nonresponders (Fig. 3B). Also, patients with moderate benefit tended to have either high TMB or low SCNAs burden, but not both (Fig. 3B). We observed three patients presenting high TMB and low SCNAs burden who did not benefit from ICIs treatment. Interestingly, in‐depth inspection of these patients revealed that one of them had prolonged benefit despite radiological progression (499 days of benefit after progression) and another presented a Large cell neuroendocrine carcinoma (LCNEC) tumor known to respond poorly to ICIs.

Furthermore, Kaplan–Meier analysis based on these same four categories indicated that patients with high TMB and low SCNAs exhibited statistically significant longer PFS than those with low TMB and high SCNAs burden, those with high TMB and high SCNAs and near statistically significant than those with low TMB and low SCNAs burden (log‐rank test, *P* = 0.006, 0.029, and 0.067, respectively) (Fig. 3C). Notably, high SCNAs burden seems to curtail benefit’s duration regardless of TMB levels, as none of the patients with outstanding response exhibited high SCNAs (Fig. 3C).

Finally, TMB and SCNAs burden were integrated as continuous variables, together with patient’s sex and ICIs’ target, in a multivariate Cox proportional hazards model stratified by histology and smoking status. This analysis indicated that both TMB and SCNAs combined are significantly associated with ICIs response even when correcting by potential confounding factors (Fig. 3D).

Importantly, similar results were obtained when performing the analysis above using patient’s overall survival (OS)—instead of PFS—as a measure of benefit (Fig. S4A, S4B).

Altogether, these data indicate the utility of both biomarkers to discriminate patients with benefit to ICIs in a cohort of patients enriched in long‐term responders.

### 3.5 Validation through analysis of publicly available data

As stated in the introduction, NSCLC long‐term responders to ICIs remain poorly molecularly characterized and indeed integrative studies using genomic and transcriptomic data are almost nonexistent. Nevertheless, we sought to validate some of our findings by reanalyzing a publicly available cohort (NSCLC‐MSK) for which genomic data (by targeted next‐generation sequencing) as well as PD‐L1 expression data (by IHC) were available [20]. Of note, both TMB and SCNAs burden (referred to as Fraction of Genome Altered (FGA) in the original manuscript) were estimated from a gene panel, which owing to the size of the fraction of the genome included in the panel and the type of genes included (mostly cancer‐related genes) is likely less accurate than WES or whole‐genome sequencing (especially when estimating SCNAs burden).

**Fig. 4:**
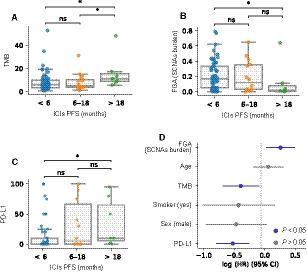
NSCLC‐MSK cohort analysis. (A) TMB, (B) FGA (SCNAs burden), and (C) PD‐L1 distribution across groups of ICIs benefit. Mann–Whitney–Wilcoxon tests have been used to determine differences between clinical benefit groups (A, B, C). ns: 0.05 < *P *≤ 1.0; *: 0.01 < *P *< 0.05. (D) Multivariate Cox proportional hazards model for FGA (SCNAs burden), age, TMB, smoking status, sex, and PD‐L1, stratified by histology. Features represented in blue are statistically significant (*P* < 0.05).

We stratified patients into the three same categories based on their PFS (no benefit, moderate, and long‐term benefit) as we did in Fig. 1B,E. As observed in our cohort, patients with long‐term benefit had substantially higher TMB than those with moderate or no benefit (MWW, *P* = 0.036 and 0.012, respectively) (Fig. 4A), while no statistically significant differences were observed between these two groups (Fig. 4A). Additionally, we observed that long‐term responders had substantially higher PD‐L1 expression (MWW, *P* = 0.013) and lower SCNAs burden (MWW, *P* = 0.023) than patients with no benefit (Fig. 4B,C).

The integrative analysis performed on our cohort (Fig. 3A) suggests the potential value of combining TMB, SCNAs burden, and PD‐L1 expression. In our cohort, we did not build such model due to the reduced number of patients with *CD274* (PD‐L1) gene expression data. Nevertheless, we could create it using the NSCLC‐MSK cohort. Thus, we incorporated TMB, SCNAs burden, and PD‐L1 expression, together with other clinically relevant features to a multivariate Cox proportional hazards model, which was stratified by tumor histology. Our analysis indicates that all three features are statistically associated with patient’s PFS (Fig. 4D).

Finally, the increased TMB and PD‐L1 levels in long‐term responders were further validated using a second independent cohort treated with PD‐1 plus CTLA‐4 blockade [14] (Fig. S5A, S5B).

Thus, the analysis of two independent datasets confirms our findings, highlighting the importance of TMB to discriminate long‐term responders. Additionally, we showed the value of combining TMB, PD‐L1, and SCNAs burden as complementary determinants of ICIs response.

## 4 Discussion

Advanced NSCLC treatment strategies have substantially changed since the emergence of ICIs. However, only a fraction of patients benefits from this type of immunotherapy, and most that do eventually acquire resistance. Thus, there is a need to find biomarkers to identify patients who will benefit from ICIs, specifically those who will present a long‐lasting response which, despite a recent publication [8], remain largely unexplored.

To this end, we gathered a new cohort of NSCLC patients treated with ICIs enriched in long‐term responders and analyzed genomic and transcriptomic features aiming to better understand the determinants of ICI‐associated long‐term response. Additionally, we validated our findings by reanalyzing public data from previous studies [14, 20].

The relevance of TMB as biomarker to predict response upon ICIs treatment is under debate [8, 15, 18, 20, 29, 30, 31]. We believe that our data shed light on this topic, as we found that the association between clinical benefit and TMB is mainly driven by patients with long‐term response (>18 months), whose TMB is much higher than the rest of patients, while we did not observe substantial differences regarding TMB between patients with no or moderate clinical benefit. This observation, originally based on our cohort, was further validated by reanalyzing two publicly available cohorts [14, 20]. Thus, in an unselected cohort, in which the proportion of individuals with long‐lasting benefit would be small, the association between TMB and ICIs response could easily go unnoticed. Our data indicate that TMB might be a biomarker of long‐term response (PFS > 18 months) rather than of durable clinical benefit (PFS > 6 months). Thus, there is a subgroup of patients who benefit from ICIs treatment (those with a PFS between 6 and 18 months), but do not exhibit higher TMB than those without benefit, a feature that seems to be specific of long‐term responders. These patients, with moderate benefit, must present other features that would explain this positive response to the treatment.

In fact, we evaluated the influence of SCNAs burden, which remains less studied particularly in advanced NSCLC. Our data highlight its importance as determinant of response to ICIs, as patients with high SCNAs burden exhibited a poorer response than the rest. In contrast, no response differences seem to exist between patients with medium or low SCNAs burden. Altogether it suggests a detrimental effect of high levels of chromosomal aberrations—presumably chromosomal instability—upon ICIs treatment, which could be at least partially explained by its negative correlation with immune infiltration as discussed below.

Furthermore, TMB and SCNAs are independent biomarkers and our data indicate the value of combining both features to discriminate those patients who will achieve sustained benefit upon ICIs treatment.

Beyond these features, other tumor genomic alterations have been reported to be indicators of response in previous studies, such as individual gene mutations and copy number alterations [29, 32, 33]. However, we could not identify a single gene associated with response or long‐term benefit after appropriate statistical correction; even though this might not be surprising as the inferred number of patients required to identify individual genes was predicted to be substantial [29]. The degree of homozygosis of human leukocyte antigen (HLA) genes and the HLA allele types has been also shown to influence ICIs response [34]. While we did study whether there was an association between HLA homozygosis and response to ICIs in our cohort, we did not observe a trend toward more heterozygosis in any group (data not shown).

We also presented gene transcription data, which indicates that T‐cell activity‐related transcriptional signatures are associated with clinical benefit to ICIs, consistent with previous reports [9, 10, 12, 13]; however, it does not seem to discriminate between moderate and long‐term response. Additionally, we identified specific immune cell‐type populations enriched in responders, some of which (CD8, natural killers, B cells) have been related to the ability of the immune system to eliminate tumor cells [35, 36]. Notably, a recent report found that PD‐L1 expression was the most reliable biomarker associated with long‐term overall survival upon PD‐1 blockade [8]. Consistent with this study, our results evaluating *CD274* (PD‐L1) gene transcript levels from a Nanostring panel indicate a similar result. Similarly, we found that patients with long‐lasting benefit following ICIs exhibited higher levels of PD‐L1 in two independent cohorts.

We also investigated the interplay between tumor‐intrinsic features and immune infiltration. It is worth highlighting that we found that immune‐related signatures negatively correlated with SCNAs burden as reported from TCGA data [19], which could explain why patients whose tumors bore high levels of chromosomal aberrations responded poorly to ICIs. In contrast to SCNAs burden and consistent with other studies [8, 11, 20], our data indicate that TMB does not correlate with immune‐related transcriptional signatures nor with PD‐L1. It is worth mentioning that *CD274* (PD‐L1) expression, in contrast to other immune‐related signatures, did not seem to correlate with SCNAs burden. Tumors may evade the immune system through different mechanisms such as expressing PD‐L1 or by preventing infiltration of immune cells within the tumor, thereby an elevated burden of SCNAs might be indicative of the latter.

Thus, both SCNAs burden and PD‐L1 expression might provide complementary information, which in combination with TMB can enhance our ability to identify patients who will exhibit sustained response to ICIs. In our cohort, we could not build a model including the three features together due to sample size limitations. Nevertheless, we could evaluate them in the NSCLC‐MSK cohort and we showed, using multivariate analysis, that the three factors are indeed significantly associated with ICIs response.

Similarly, the relatively low number of patients with expression data limits our capacity to discriminate between medium and long‐term benefit and curtails the use of this data in survival analyses (i.e., multivariate model). Thus, we could not assess the value of combining these gene transcription‐based scores together with TMB to better identify patients with long‐term benefit.

Another limitation of our study is that we could not evaluate intra‐tumoral heterogeneity within the primary tumor [37], as well as possible differences between primary tumor and metastases. Certainly, analysis of multiple sites of the same primary tumor and/or metastases would provide a higher accuracy when estimating TMB, SCNAs burden, and tumor immune infiltration. However, obtaining these samples is undoubtedly challenging in advanced NSCLC patients. Additionally, WES might not be the best approach to determine SCNAs along the genome; hence, other techniques such as low pass whole‐genome sequencing could offer more accurate results. Finally, our cohort is strongly enriched in patients with long‐term benefit upon ICIs treatment, allowing a detailed study of this subgroup, which was the primary aim of our study. Therefore, our cohort does not fully recapitulate the clinical reality in advanced NSCLC, as in a cohort of unselected patients, long‐term ICIs responders would be few. Nevertheless, we validated our findings by analyzing two independent cohorts with publicly data available.

## 5 Conclusion

Our data indicate that high TMB moderately associates with durable clinical benefit to ICIs, while it is strongly correlated with long‐term response. Conversely, high SCNAs burden is indicative of lack of response. Additionally, patients who benefit from ICIs treatment present higher levels of immune infiltration signatures, even though this does not seem suggestive of benefit’s duration. Additionally, *CD274* expression is particularly high in long‐term responders. TMB is independent of SCNAs burden or tumor immune infiltration, which are negatively correlated. Combining TMB and SCNAs burden allows to discriminate patients with ICI‐associated long‐term benefit (either PFS or OS) better than each feature individually. Notably, our observation that TMB is strongly enriched in long‐term responders to ICIs was validated in two independent cohorts, suggesting that TMB might be a biomarker of long‐lasting benefit rather than durable clinical benefit (6 months of PFS). Finally, using the NSCLC‐MSK cohort, we described that TMB, SCNAs burden, and PD‐L1 are significantly associated with clinical benefit following ICIs treatment.

## Conflict of interest

EF reports the following conflicts of interest: advisory role or speaker’s bureau: AbbVie, AstraZeneca, BerGenBio, Blueprint medicines, Boehringer Ingelheim, Bristol‐Meyers Squibb, Celgene, Eli Lilly, Guardant Health, Janssen, Medscape, Merck KGaA, Merck Sharp & Dohme, Novartis, Pfizer, priME Oncology, Roche, Samsung, Takeda, Touchtime. Board: Grifols, independent member. Research funding: Fundación Merck Salud, Grant for Oncology Innovation EMD Serono. AV reports advisory role: Sysmex, Novartis, Merck, Roche, Bristol‐Meyers Squibb, Guardant Health, and research funding: Bristol‐Meyers Squibb, Novartis, Debio, Sysmex, Cellestia Biotech, Roche. PN has consulted for Bayer, Novartis, MSD, and Targos, and received compensation. IS reports advisory role, speaker’s bureau or travel compensation: Roche Farma, Abbvie, Roche Diagnostics, Merck Sharp & Dohme, Pfizer, Takeda, Bristol‐Myers Squibb, Lilly, Sysmex, Boehringer Ingelheim. CC has been partially supported by Grant for Oncology EMD Serono research funding to EF. AMM provided consultation, attended advisory boards and/or speaker's bureau for the following organizations: BMS, Roche, MSD, Pfizer, Boehringer Ingelheim, AstraZeneca. AN reports advisory role, speaker’s bureau or travel compensation: Bristol‐Myers Squibb, F. Hoffmann La Roche AG, Pfizer, Boehringer Ingelheim, Oryzon Genomics, Merck Sharp & Dohme. SC Bristol‐Myers Squibb Recipient F, Hoffmann La Roche AG, Pfizer, Boehringer Ingelheim, MSD Oncology, Amphera. AC reports advisory role and/or travel compensation: Bristol‐Myers Squibb Recipient, F. Hoffmann La Roche AG, Pfizer, Boehringer Ingelheim, MSD Oncology, Kyowa Kirin, Celgene. PI reports advisory role and/or travel compensation: Bristol‐Myers Squibb Recipient, F. Hoffmann, La Roche AG, Merck Sharp & Dohme, Boehringer Ingelheim, MSD Oncology, Rovi, Yowa Kirin, Grunenthal Pharma S.A., Pfizer. NP reports advisory role and/or travel compensation: Bristol‐Myers Squibb Recipient, F. Hoffmann La Roche AG, Pfizer, Boehringer Ingelheim. AP reports advisory role and/or travel compensation: Pfizer, Novartis, Roche, MSD Oncology, Lilly, Daiichi Sankyo, Amgen, Boehringer, PUMA, Oncolytics Biotech, Abbvie, Nanostring Technologies. All remaining authors have declared no conflicts of interest.

## Author contributions

Joan Frigola involved in conceptualization, data curation, formal analysis, methodology, visualization, writing—review and editing, software. Alejandro Navarro involved in resources, data curation, writing—review and editing. Caterina Carbonell involved in resources, investigation, writing—review &and editing. Ana Callejo involved in resources, writing—review and editing. Patricia Iranzo involved in resources, writing—review and editing. Alex Martinez‐Marti involved in resources, writing—review and editing. Nadia Saoudi‐Gonzalez involved in resources, writing—review and editing. Nuria Pardo involved in resources, writing—review and editing. Susana Cedrés involved in resources, writing—review and editing. Debora Martinez involved in investigation, writing—review and editing. Jose Jiménez involved in resources, writing—review and editing. Irene Sansano involved in resources, writing—review and editing. Francesco M Mancuso involved in data curation, formal analysis, writing—review and editing. Paolo Nuciforo involved in resources, writing—review and editing. Luis M Montuenga involved in writing—review and editing and formal analysis. Montserrat Sánchez‐Cespedes involved in writing—review and editing, formal analysis. Aleix Prat involved in writing—review and editing, resources, investigation. Ana Vivancos involved in investigation, writing—review and editing, resources. Enriqueta Felip involved in conceptualization, funding acquisition, supervision, writing—review and editing. Ramon Amat involved in conceptualization, investigation, supervision, writing—original draft, writing—review and editing.

## Supporting information

Fig S1. Patients’ TMB distribution across clinical features.
Fig S2. Expression profiling of tumor microenvironment.
Fig S3. Correlations between features.
Fig S4. Overall survival analysis.
Fig S5. Cancer Cell validation cohort analysis.

Table S1. Cohort clinical data summary.

Table S2. Patient information and clinical outcome.

Table S3. Gene list for gene expression signatures.

Table S4. Mutations per patient.

Table S5. Copy number alterations per patient.

Supplementary Material
