# Supplementary figures and images for: Molecular profiling of long‐term responders to immune checkpoint inhibitors in advanced non‐small cell lung cancer
Source: Mol Oncol. 2021 Jan 6;15(4):887–900. doi: 10.1002/1878-0261.12891 (PMC8024716; doi:10.1002/1878-0261.12891)

Figure S1

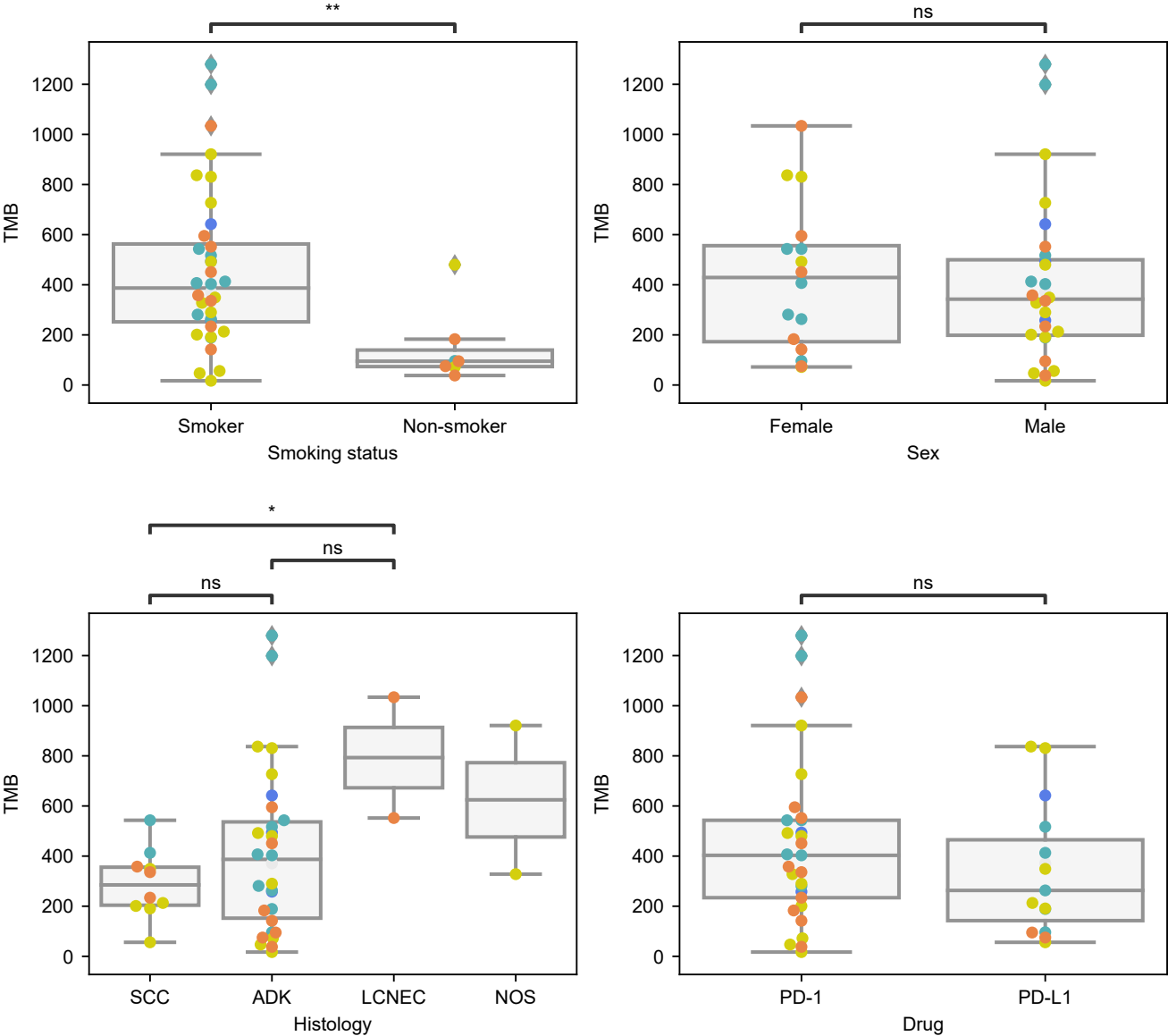

Figure S2

A

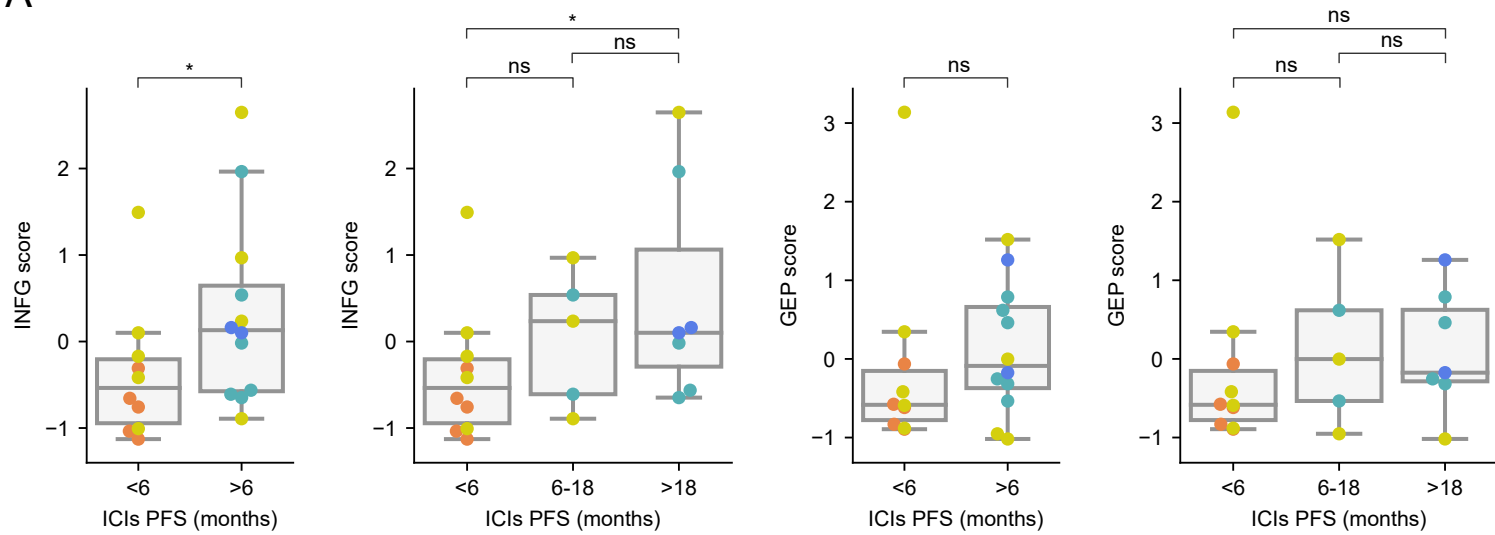

B

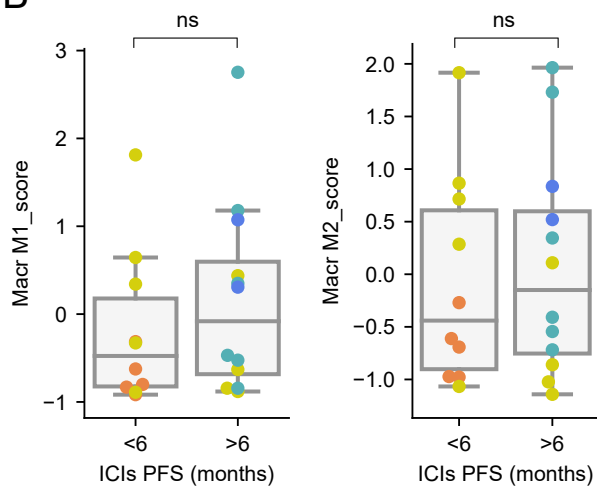

C

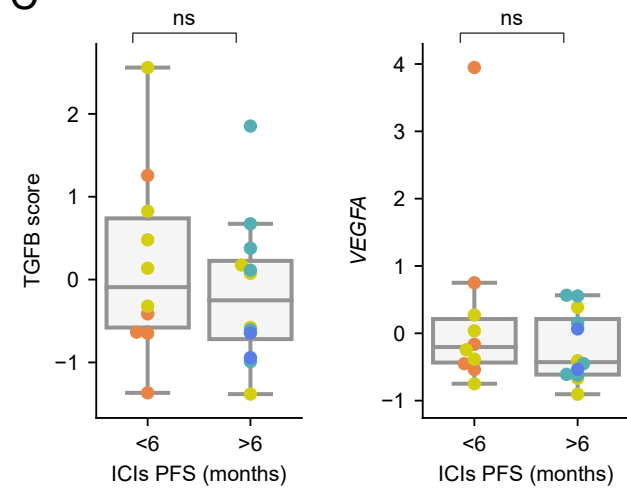

D

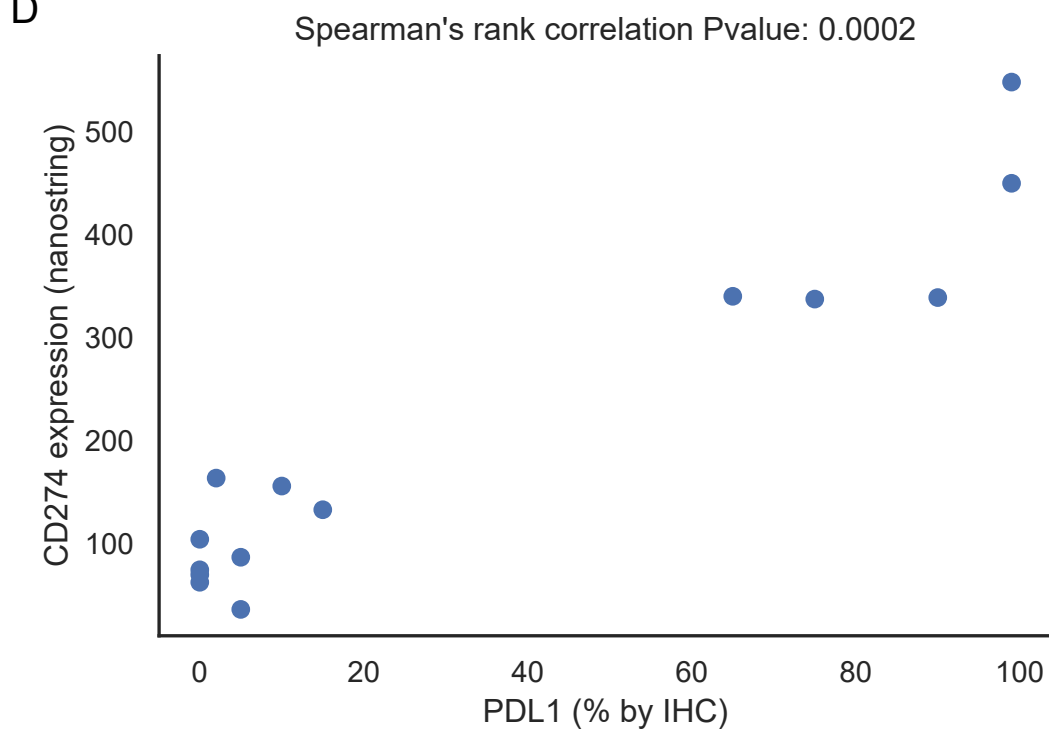

Figure S3

A

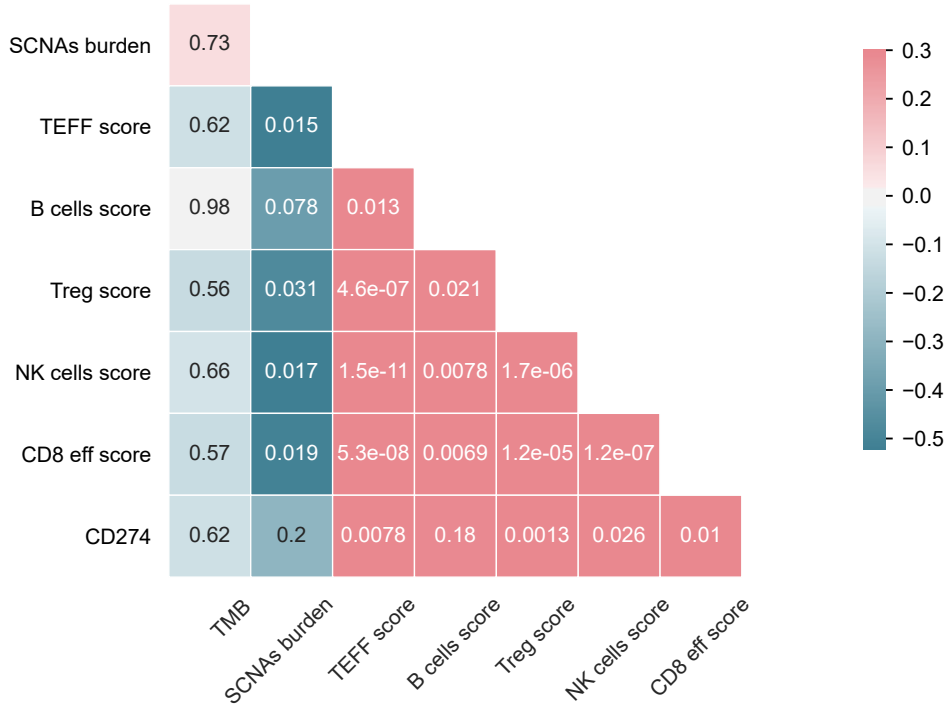

B

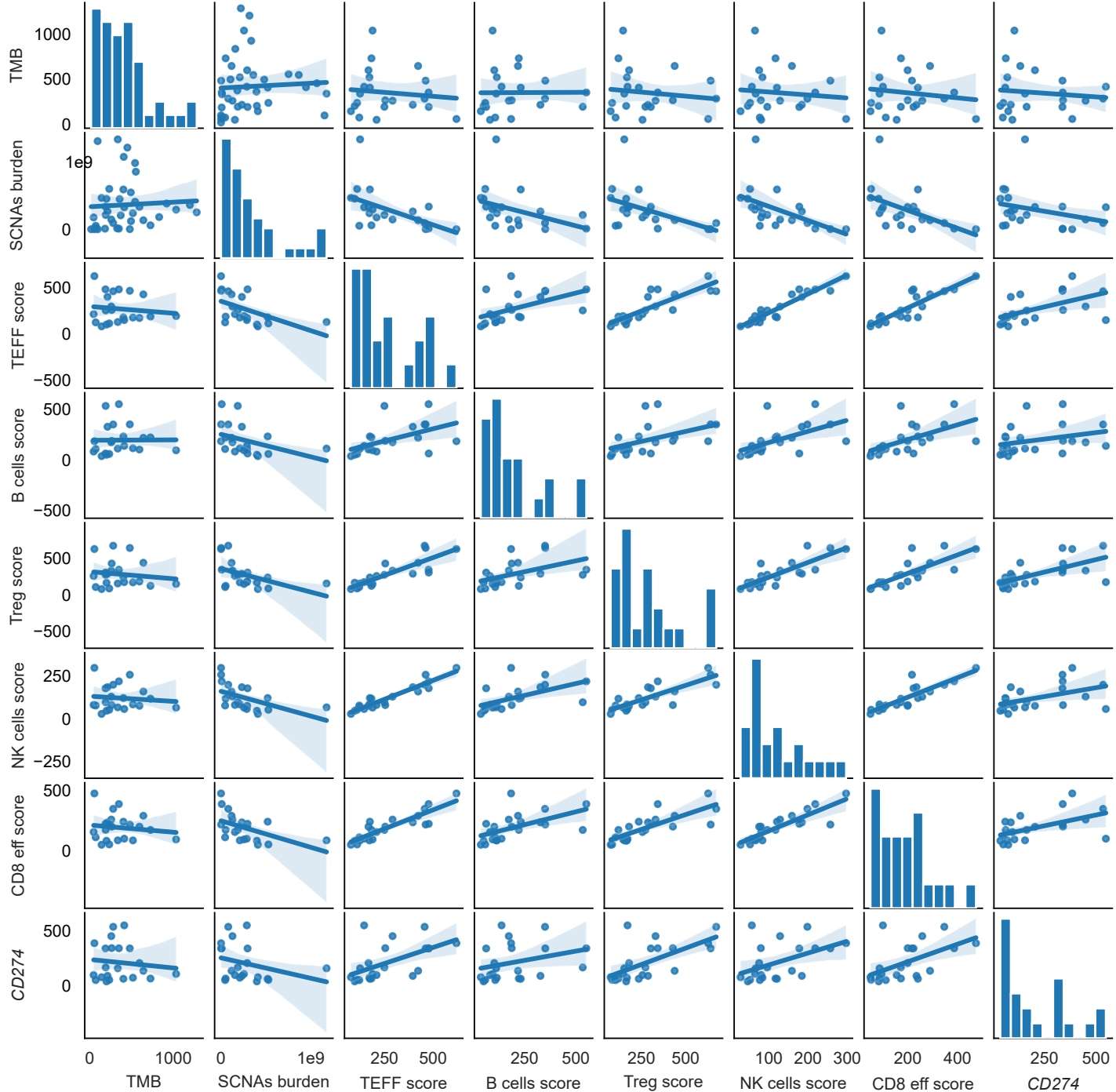

Figure S4

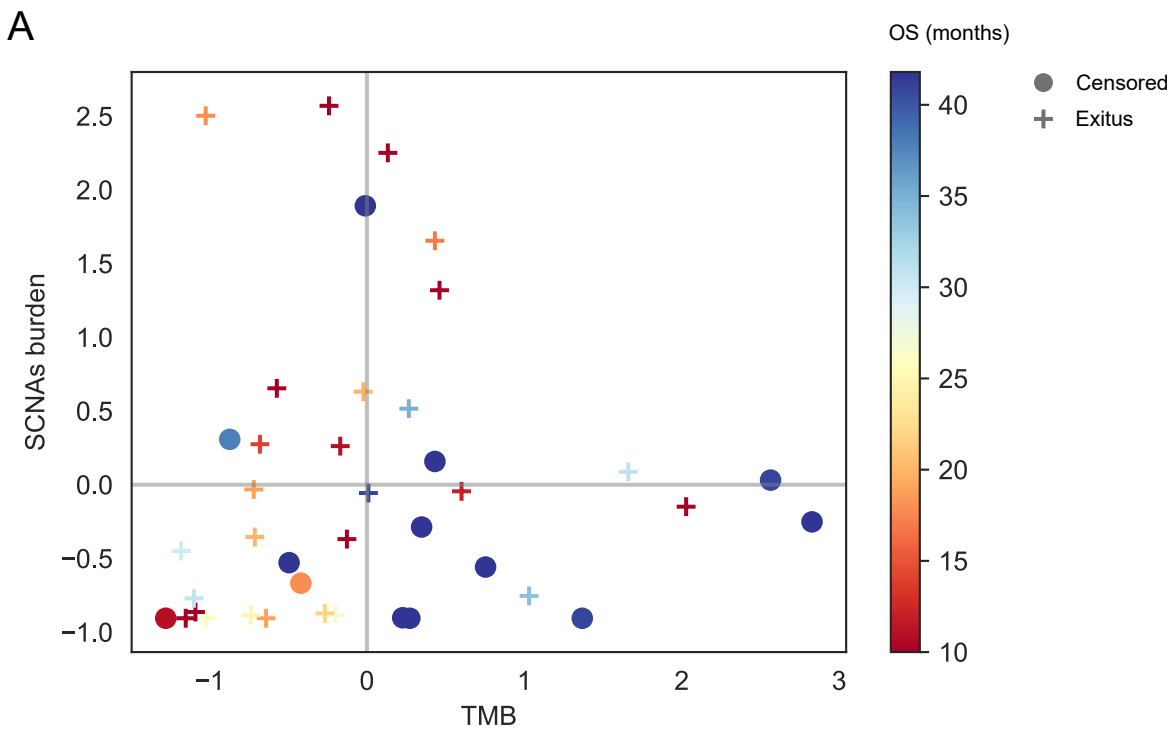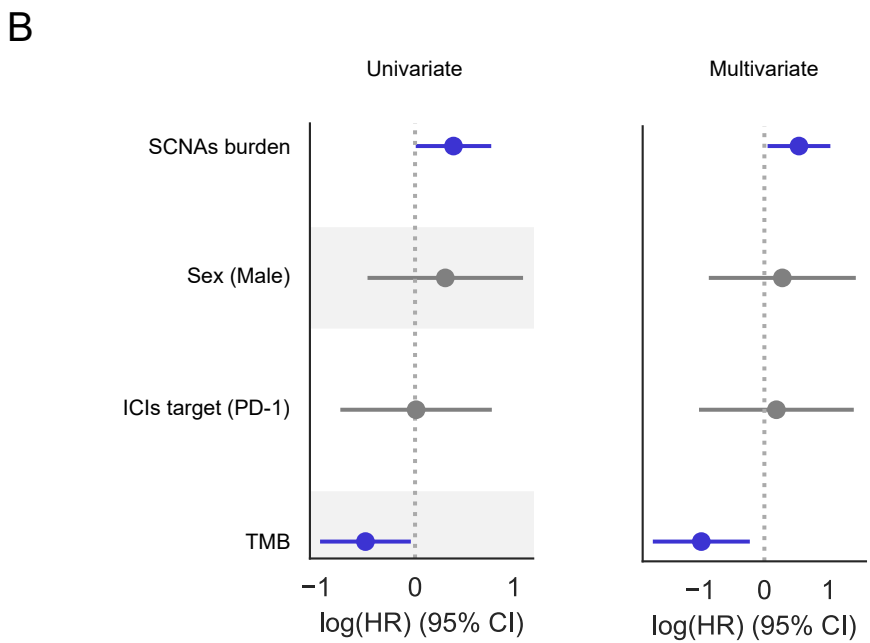

Figure S5

A

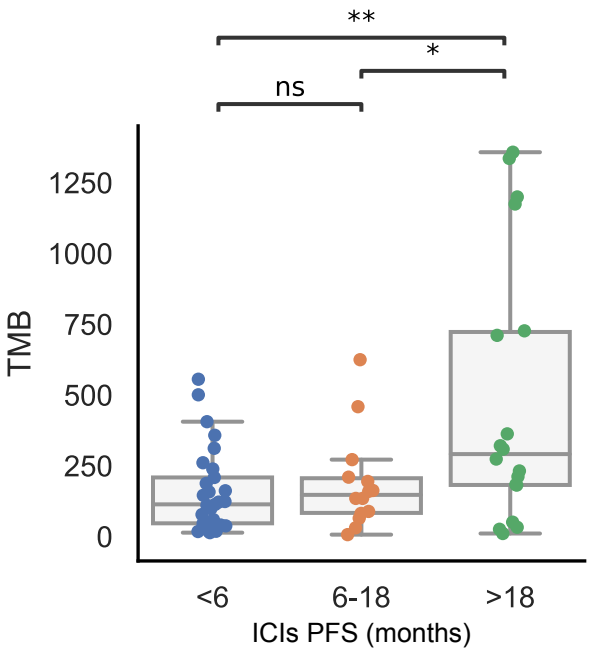

B

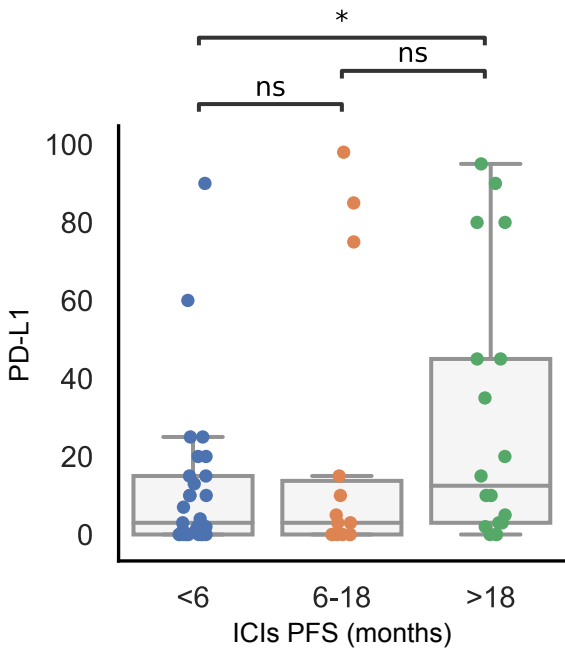

Supplement: Supplementary file 1 — Fig S1. Patients’ TMB distribution across clinical features. Fig S2. Expression profiling of tumor microenvironment. Fig S3. Correlations between features. Fig S4. Overall survival analysis. Fig S5. Cancer Cell validation cohort analysis. [file MOL2-15-887-s003.pdf]
