# Supplementary material for: Molecular profiling of long‐term responders to immune checkpoint inhibitors in advanced non‐small cell lung cancer
Source: Mol Oncol. 2021 Jan 6;15(4):887–900. doi: 10.1002/1878-0261.12891 (PMC8024716; doi:10.1002/1878-0261.12891)
Supplement: Supplementary file 7 — Supplementary Material [file MOL2-15-887-s005.docx]

**Supplementary figure legends.**

**Supplementary Figure 1. Patients’ TMB distribution across clinical features**. TMB distribution across patients grouped by different clinical features. Color indicates best response to treatment (CR: Complete Response, PR: Partial Response, SD: Stable Disease and PD: Progressive disease). Mann-Whitney-Wilcoxon tests have been used to determine differences between groups. ns: 0.05 < p <= 1.0; *: 0.01 < p <0.05; **: 0.001 < p <= 0.01.

**Supplementary Figure 2. Expression profiling of tumor microenvironment**. (A) INFG, GEP signature scores across groups of benefit to ICIs treatment. (B) M1 and M2 macrophages signature scores distribution across groups of benefit to ICIs treatment. (C) TGFB score and *VEGFA* expression distribution across groups of benefit to ICIs treatment. Color indicates best response to the treatment (CR: Complete Response, PR: Partial Response, SD: Stable Disease and PD: Progressive disease). Mann-Whitney-Wilcoxon tests have been used to determine differences between ICIs benefit groups. ns: 0.05 < p <= 1.0; *: 0.01 < p <0.05; **: 0.001 < p <= 0.01. (D) Spearman’s rank correlation between *CD274* expression (Nanostring) and PD-L1 expression (immunohistochemistry).

**Supplementary Figure 3. Correlations between features.** (A) Pearson correlations between TMB, SCNAs burden, TEFF score, indicated immune cell type signature and *CD274* expression. Color represents the correlation coefficient. The P value of each correlation is shown within each cell. (B) Correlogram between all features included in panel A.

**Supplementary Figure 4. Overall survival analysis.** (A) Scatter plot representation of all samples in the cohort for which TMB and SCNAs burden has been computed. X axis corresponds to TMB and Y axis to SCNAs burden. Horizontal and vertical gray lines represent the SCNAs burden and TMB means respectively. Patients have been colored based on their Overall Survival (months). Patients represented as a + sign are those that passed away as a result of their cancer disease, while those represented as a dot are patients censored in the survival analysis, either because they are still ongoing or for any other reason. (B)  Univariate and multivariate Cox proportional hazards model of SNCAs burden, ICIs target, Sex and TMB. Multivariate analysis has been stratified by histology and smoking history of the patients. The event considered in this model is patient’s death, and the time to event is their overall survival since the beginning of ICIs treatment. Features represented in blue are statistically significant (p < 0.05).

**Supplementary Figure 5. Cancer Cell validation cohort analysis.** (A) TMB, (B) PD-L1 distribution across groups of ICIs benefit. Mann-Whitney-Wilcoxon tests have been used to determine differences between ICIs benefit groups. ns: 0.05 < p <= 1.0; *: 0.01 < p <0.05; **:0.001 < p <0.01.
